# Can work-to-family conflict lead to preschool children’s social behavior problems?—The chain mediating roles of guilt about parenting and parent-child relationships

**DOI:** 10.3389/fpsyg.2023.1195994

**Published:** 2023-07-06

**Authors:** Yan Wang, Dasheng Shi, Guolei Liu, Mengmeng Zhang, Xinhong Zheng

**Affiliations:** ^1^School of Education, Minzu University of China, Beijing, China; ^2^Department of Education, Hebei Institute of International Business and Economics, Qinhuangdao, China; ^3^School of Education, Beijing Normal University, Beijing, China; ^4^Psychological Crisis Intervention Center of PLA 984 Hospital, Beijing, China

**Keywords:** preschool children, work-to-family conflict, social behavior problems, guilt about parenting, parent-child relationships

## Abstract

Parents’ work-to-family conflict has been reported to be associated with preschool children’s social behavior problems, but the underlying mechanisms of this association in the Chinese cultural context remain unclear. Based on ecosystem theory and the spillover-crossover model theory of emotion, this study aimed to examine the correlation between parents’ work-to-family conflict and preschool children’s social behavior problems in China, as well as the mediating role of guilt about parenting and parent–child relationships. Structural equation modeling was used to check the research hypotheses with a sample of 3,038 parents of Chinese preschool children. The main findings of this study are as follows: (1) Work-to-family conflict faced by parents was positively associated with guilt about parenting and preschool children’s social behavior problems; (2) The effect of guilt about parenting on preschool children’s social behavior problems was bidirectional; guilt about parenting was positively related to preschool children’s social behavior problems, but when guilt about parenting prompted parents to adjust their parent–child relationships, it was negatively related to preschool children’s social behavior problems. Taken together, these results further explain the interaction between parents’ work-to-family conflict and preschool children’s social behavior problems and discuss the influence of multiple factors on preschool children’s social behavior problems. Theoretically, this study enriches the theoretical basis of the interaction with resources from the external environment of home education and family education. Practically, it implies that multiple levels, such as the government, early childhood education institutions, and work units, should give more support to preschool children’s family education and thus work together to promote the healthy development of preschool children.

## Introduction

Behavior problems are a manifestation of children’s social maladjustment, mainly referring to immature behaviors in children’s emotional and social adjustment (Achenbach et al., 1987), including both explicit behavior problems (e.g., aggression and defiant behavior) and implicit behavior problems (e.g., depression/anxiety and social withdrawal; Achenbach, 1991). There are growing prospective evidence that behavior problems identified in the preschool stage ^1^ often persist and bring negative effect on the future development of the individual (Campbell, 1995; Liang et al., 2022). To illustrate, children who manifest markedly disruptive behavior problems during early adolescence typically have a preceding history of problems that originated during their preschool stage (e.g., Loeber and Dishion, 1983; Moffitt, 1990; Campbell, 1995). Furthermore, social adjustment problems in early childhood can also predict, to some extent, future problems such as lower learning ability and lower academic achievement (Bulotsky-Shearer and Fantuzzo, 2011). It is evident that early childhood behavior problems significantly impact individuals’ future development (Egeland et al., 1990; Campbell, 1994; Campbell, 1997; Winsler et al., 2000). As such, researchers need to focus on examining risk and protective factors associated with young children’s behavior problems. This study takes a multi-perspective approach, exploring the influences of various factors on preschool children’s behavior problems, including educational background and parenting behaviors. This current study aims to identify effective strategies for reducing children’s behavior problems, enhancing their social adjustment abilities, and promoting their healthy physical and mental development.

“Social skills are defined as abilities needed to perform competently in a social situation, including encoding skills (e.g., perception and interpretation of a situation), decision skills (e.g., social self-efficacy), and enactment skills (e.g., asking a friend to get together, planning activities with friends; Cavell, 1990; Devine et al., 2012).” Social skills are the basis for children’s social adjustment since they are likely to help improve the quality and effectiveness of children’s social interactions and social adjustment, which in turn, lead to less behavior problems (Winsler and Wallace, 2002). A variety of factors have been proposed to influence the course of early social skill acquisition; however, for young children who just started their learning in kindergarten, such skills are likely dependent on the family context (Anthony et al., 2005).

According to the ecosystem theory (Bronfenbrenner, 1993), family is the innermost system that influences individuals’ development, called micro-system. Children first acquire social skills through direct contact with family members. Therefore, the family environment exerts strong influence on children’s early development that is based on social skills. Specifically, some factors (e.g., family social status, family member relationships, marital status and quality) may influence preschool children’s acquisition of social skills and affect their likelihood of social behavior problems. That has been confirmed by numerous studies (e.g., Campbell and Cluss, 1982; McGee et al., 1984; Dadds and Powell, 1991; Koot, 1993). Family is a basic unit that constitutes the ecosystem for children’s development, the development of children is influenced not only by multiple factors within the family, but also by related factors outside the family. In modern, a prominent example of the ecosystem is the parents’ workplace. The spillover-crossover model suggests that individuals’ emotions, attitudes, and behaviors in the work domain can spill over into the family domain and influence the emotions, attitudes, and behaviors of their family members through social interaction (Bakker and Xanthopoulou, 2009; Ilies et al., 2017). The characteristics of the workplace, such as the working hours, intensity, and input-reward ratio, are likely to influence children’s development, mainly through its impact on family processes and family functioning (Bronfenbrenner, 1986; Hess and Pollmann-Schult, 2019). Therefore, research on the family education of preschool children should not be limited to the family; rather, it should be extended to include external factors such as sociocultural and work characteristics, and how they are correlated to the development of children’s social skills.

With the ongoing economic transformation and urbanization in China, significant changes in the public’s lifestyle have emerged, leading to a direct impact on family education of preschool children. Notably, the increasing number of dual-income families in urban areas has placed a significant burden on parents, especially mothers, who must balance work responsibilities with household and family education duties to ensure adequate income for their families (Tong and Liu, 2010). Additionally, changes in urban living patterns have resulted in more nuclear families with children, making it crucial for parents to assume more responsibility for family education, rather than relying on the children’s grandparents (Ma, 2023). However, traditional gender concepts continue to influence preschool children’s home education. Although dual-income families require a cooperative model in which both partners share the responsibilities of family education, traditional ideas surrounding gender roles still motivate mothers to take on the primary responsibility for family education. This poses challenges for young Chinese mothers, including maintaining a work-family balance, fostering positive emotions in family education, and achieving happiness in life (Tong and Liu, 2010).

Given the challenges faced by working parents in balancing family and work, this study aims to explore the factors that mediate the relationship between work-family factors and children’s social adjustment problems. Notably, there is a scarcity of empirical research on the association between parents’ workplace and children’s development (Vieira et al., 2016), including the internal mechanism of this relationship, particularly within the Chinese cultural context. Therefore, this study focuses on examining the variable of Chinese parents’ experiences in managing their work and family roles and explores the mechanism of action in relation to preschool children’s social behavior problems.

### Work-to-family conflict (WFC) and preschool children’s social behavior problems

Many parents of preschool children are experiencing some degree of stress or anxiety related to the attempt to balance the conflicting demands of work and family (Duxbury and Higgins, 1994). Work–family conflict (WFC) is “a form of inter-role conflict in which the role pressures from the work and family domains are mutually incompatible in some respect. That is, participation in the work (family) role is made more difficult by virtue of participation in the family (work) role” (Greenhaus and Beutell, 1985). Work–family conflict (WFC) manifests in two distinct ways. The first is through the interference of work responsibilities with family life, referred to as work-to-family conflict (WFC). The second involves family responsibilities diminishing the efficacy of work, known as family-to-work conflict (FWC). Drawing on Pearlin et al.'s (1990) stress process model and Bronfenbrenner (1986) ecological model of human development, research suggests that work-to-family conflict is primarily associated with adverse outcomes for children. This is due to the demands of the workplace, which limit the time and energy parents have available for their families, impede the flexibility and autonomy of family life, and negatively impact family education practices. Facing with fierce job competition, pressure of career development, and financial pressure due to the high cost of childcare, work-to-family conflict are common among Chinese young parents, which is very likely to have an impact on family life, especially on childcare. Zhang and Lin (2020) have conducted empirical studies in the Chinese context, showing that the extended time of both family and work and the double demands of emotional investment lead to the “time shortage” of urban young parents, which further increases the rearing anxiety of the family emotional atmosphere. Lau (2009) examined the impact of work-to-family conflict on the quality of parent–child interactions in Hong Kong, indicated that parents’ work-to-family conflict negatively affected the quality of parent–child interactions, which in turn caused harm to children’s self-esteem. Song et al. (2023) also found that Parents’ work–family conflict not only directly predicts children’s social behavior problems, but also indirectly increases it through the stress about parenting. Specifically, studies have indicated that children whose parents experience higher levels of work-to-family conflict tend to exhibit higher levels of emotional symptoms, conduct problems, hyperactivity/inattention, and peer relationship problems (Dinh et al., 2017; Chai and Schieman, 2021; Song et al., 2023). Based on the above research findings, the research hypothesis 1 of this study is proposed, which is that there is a positive correlation between the level of work-to-family conflict experienced by parents and preschool children’s social behavior problems.

### Work-to-family conflict, guilt about parenting and preschool children’s social behavior problems

Guilt about parenting has been suggested as a potential mediating variable between work-to-family conflict and social behavior problems among preschool-aged children (Slobodin et al., 2020). This cognitive-emotional response occurs when parents believe that their thoughts, feelings, or behaviors as parents have caused harm to their children, leading to negative self-evaluations (Haslam et al., 2019). Guilt about parenting often arises from the competing demands of work and family responsibilities. Work-to-family conflict not only increases parents’ daily stress and anxiety, but also creates a perception that their careers impede their caregiving role, potentially causing harm to their children (Dinh et al., 2017). Many studies have shown that work-to-family conflict may trigger guilt about parenting (e.g., Livingston and Judge, 2008; Bosman, 2021). Zhang (2002) suggests that traditional Chinese family values and patterns increase the likelihood that work-to-family conflict lead to guilt about parenting. For example, if the mother is on a business trip and the father has to take the children to eat fast food, the traditional concept of family will think that this family lacks rules and is irresponsible to the children. Zhang (2002) further believes that modern professional families need to have new standards of home-work relationships, thereby reducing guilt about parenting. Parents of preschool children tend to experience guilt more frequently than non-parents or parents of older children (Borelli et al., 2016; Korabik, 2017), with guilt about parenting being a significant component of work-family guilt for this group.

There could exist an association between guilt about parenting and social behavior problems in preschool children. As noted earlier, guilt about parenting is a negative emotional response associated with work-to-family conflict, which may pose a risk to the children’s physical and mental well-being (Jocson and McLoyd, 2015). At the same time, as a typical moral emotion, guilt is a negative affective experience that individuals feel after violating moral standards, desirable norms, or moral rules. It motivates individuals to make up for the harm they have caused to others. Parents may engage in more compensatory behaviors driven by guilt, such as adopting overly permissive parenting styles, increasing parent–child interaction, or overindulging children, in an effort to alleviate their guilt and make up for perceived harm done to their children (Nomaguchi and Milkie, 2003; Martínez et al., 2011; Cho and Allen, 2012). These compensatory behaviors may have new effects on children’s social behaviors. In summary, it is reasonable to propose that work-to-family conflict leads to guilt about parenting, which in turn influences the development of social behavior problems in preschool children. Based on the above analysis, this study proposes the hypothesis 2: which is that guilt about parenting mediates the relationship between work-to-family conflict and preschool children’s social behavior problems.

### Work-to-family conflict, parent–child relationships and preschool children’s social behavior problems

Many studies have demonstrated the existence of an indirect or mediated relationship between work-to-family conflict experiences and children’s well-being (Galambos et al., 1995; Crouter et al., 1999). According to mother-infant attachment theory (Bowlby, 1979), the parent–child relationships may be a significant mediating variable between work-to-family conflict and preschool children’s social behavior problems. Parent–child relationships is the earliest form of interpersonal relationships for children, which is formed in the process of interaction between parents and children (Driscoll and Pianta, 1992; Liang et al., 2022). Previous studies found that negative parent–child relationships in early childhood are an important risk factor for anxiety problems and play an important mediating role between the parents’ own characteristics, parenting behaviors, and early childhood development (Bradford et al., 2016). First，how parents balance their work and family roles is related to the quality of their parent–child relationships (Vieira et al., 2016). Second, a good nurturing relationship between parent and children shapes social, cognitive, and emotional development of children (Antonucci et al., 2004; Popov and Ilesanmi, 2015). Conversely, negative parent–child relationships are likely to increase preschool children’s social behavior problems. Negative parent–child relationships lead to less child supervision, more punitive discipline, and less child involvement; these can further lead to children’s antisocial behaviors (Johnston et al., 1998; Aucoin et al., 2006). Many studies have explored the influence of parent–child relationships on children’s social development in the Chinese cultural context. An example of relevant research is provided by Zhang et al. (2008), who found that early exposure to high-quality parent–child relationships can help reduce adjustment problems in children. In contrast, children who experience negative and conflicting relationships with their parents tend to exhibit more disruptive and aggressive behaviors (Lyons-Ruth, 1996). Generally, it can be reasonably postulated that work-to-family conflict may serve as a negative predictor of parent–child relationships, which, in turn, have an impact on the social behaviors of preschool children. Therefore, parent–child relationships act as a mediating variable between work-to-family conflict and the social behaviors of preschool children. This constitutes the research hypothesis 3 of the present study.

### Guilt about parenting and parent–child relationships

Researches have shown that guilt about parenting can predict the parent–child relationships from two distinct perspectives. On the one hand, some scholars posit that guilt about parenting increases parental anxiety and stress, thereby hindering the development of a positive parent–child relationships (Livingston and Judge, 2008; Zeng et al., 2021). On the other hand, other scholars view guilt as a positive emotion, suggesting that guilt about parenting can motivate working parents to engage in more interactive behaviors with their children in the face of work-to-family conflict (Cho and Allen, 2012). This study lends more support to the latter perspective, proposing that guilt can have constructive properties for the parent–child relationships, and that it may weaken the negative relationship between work-to-family conflict and parent–child relationships.

Collectively, work-to-family conflict triggers feelings of guilt among parents about their family life, especially in regard to child-rearing, and this guilt may drive parents to reflect on and commit to strengthening the parent–child relationships, which in turn influences the behavioral performance of preschool children. Therefore, it can be predicted that the negative impact of work-to-family conflict on the social behavior of preschool children in China may be mediated through guilt about parenting and the parent–child relationships. This constitutes the research hypothesis 4 of this study.

## The present study

**Figure 1:**
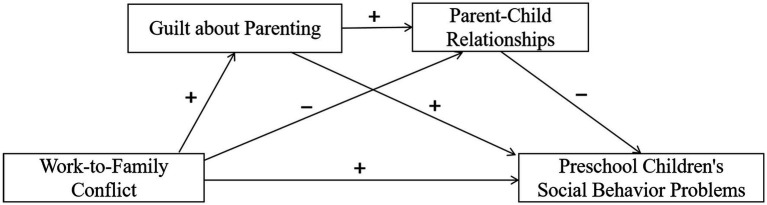
Hypothesized model of serial-multiple mediation of guilt about parenting and parent-child relationships in the relationship between work-to-family conflict and preschool children’s social behavior problems.

Building upon prior research investigating the association between parents’ work-to-family conflict and preschool children’s social behavior problems, this study aims to investigate the mediating role of guilt about parenting and parent–child relationships in this association within the unique Chinese economic and cultural context. Guided by the ecosystem of child development theory, spillover-crossover model of emotion, and attachment theory, we proposed a serial mediation model that elucidates the intricate connections between these four variables and formulated four research hypotheses (see Figure 1).

The context of child development, according to Bronfenbrenner’s ecosystem theory, consists of four distinct concentric systems: micro, meso, exo, and macro. Although the child does not directly encounter the exosystem, it impacts his development. For example, parents’ work schedules are a part of the exosystem, which may affect the amount of time parents spend with their children and influence the opportunities for parents to communicate and collaborate with schools, consequently affect the children’s development (Krishnan, 2010). Therefore, this study proposed the hypothesis that:

*Hypothesis 1*: Parents’ work-to-family conflict positively and significantly predicts preschool children’s social behavior problems.

The spillover-crossover model brings together employees’ main life domains: work and family. This theory suggests that employees’ work experiences impact behaviors, thoughts and feelings in the home domain (Rodríguez-Muñoz et al., 2013). Many scholars have been guided by this theory to study individuals’ work–family conflicts, and they have found that guilt is a common emotion experienced by individuals in work–family conflicts (e.g., Gilbert et al., 1981; Shimazu et al., 2009). For parents of preschool children, the pressure for mothers to have large quantities of face time with their children is at its apex (Milkie et al., 2015), consequently work-to-family conflict often leads to parents’ guilt about parenting. Guilt about parenting may have important intra- and inter-personal consequences (Borelli et al., 2016). For instance, parents riddled with guilt about parenting may engage in more repair behaviors, such as permissive parenting, which can have a impact on children’s social development (Nomaguchi and Milkie, 2003; Martínez et al., 2011). It can therefore be hypothesized that:

*Hypothesis 2*: Guilt about parenting mediates the association between Chinese parents’ work-to-family conflict and their preschool children’s social behavior problems. That is, work-to-family conflict may be a positive predictor of guilt about parenting, which in turn may influence the development of preschool children’s social behavior problems.

Bowlby claimed that the only initial means of communication between infant and mother is through emotional expression and its accompanying behaviors (Bowlby, 2008). It has been found that mindful parents are more involved in their children’s lives and more inclined to be aware of their children’s needs, which helps form better parent–child relationships and build secure mother-infant attachments (Siu et al., 2016). However, work-to-family conflict prevents the parent from fulfilling the demands of his/her family role. Work-to-family conflict leads to parents’ inability to give more attention and sensitivity to children, which negatively affects the parent–child relationships. Based on the parent–child relationships, the children develop an “internal working model” of social interaction (Bowlby, 1982; Ainsworth, 1989). This internal working model directly influences the style of children’s interactions with others and the development of their social competence. On the basis of the reasoning and findings, we predict that:

*Hypothesis 3*: The parent-child relationships mediate the association between Chinese parents’ work-to-family conflict and their children’s performance of social behavior problems. That is, parents’ work-to-family conflict may be a negative predictor of the parent–child relationships, carrying on to lead to preschool children’s social behavior problems.

Taken together, Hypothesis 2 and 3 suggest that:

*Hypothesis 4*: Parents’ work-to-family conflict will influence the development of preschool children’s social behavior problems through the serial mediators of guilt about parenting and parent–child relationships. That is, work-to-family conflict may be a predictor of parents’ guilt about parenting, thus significantly affects parent–child relationships, which in turn predict more preschool children’s social behavior problems.

## Methods

### Participants

The study aims to examine the association between parents’ work-to-family conflict and preschool children’s social behavior problems in economically developed coastal areas in China. A combination of purposive and convenience sampling methods was used to collect data from parents of children aged 3–6 years old between September 1 and 25, 2022. Participants were recruited from nine cities in Guangdong Province, including Zhuhai, Guangzhou, Zhanjiang, Shenzhen, Zhongshan, Foshan, Dongguan, Jieyang, and Shantou. In order to improve the efficiency of data collection and to ensure the quality of the questionnaire, we invited the directors of kindergartens in each of the above cities to participate in this study. The questionnaires were disseminated to parents of preschool children by the directors through an online platform. In order to minimize the impact of external pressure on participants’ responses, the questionnaires were designed to be anonymous. Furthermore, prior to collecting the questionnaires, informed consent was obtained from all participants. This study retained 3,197 parent questionnaires, of which, 3,038 were valid (Table 1). Samples exhibiting consistent response tendencies or response times of less than 5 minutes were considered invalid samples. Among the valid participants, 2,448 were female respondents (mothers,80.6%) and 590 were male respondents (fathers,19.4%); 816 respondents (26.9%) had the only child and 2,222 respondents (73.1%) had more than one child; 576 respondents (19.0%) had a high school education; 828 respondents (27.3%) had a junior college education; and 1,391 respondents (45.8%) had a bachelor’s degree, 243 respondents (8.0%) had a master’s degree or higher; Nearly 63.4% of parents (1,925) take care of children with the help of grandparents, 36.6% of parents (1,113) raise children completely independently without the help of grandparents. All measurements and procedures were permitted by the Institutional Review Board (IRB) of the first author’s institution.

### Measures

The research instrument consisted of four components: the Work–Family Conflict Scale, the Social Competence and Behavior Evaluation Scale, the Guilt about Parenting Scale, and the Child Adjustment and Parent Efficacy Scale. The reliability and validity of the respective scales in their Chinese versions have been verified.

### Work–family conflict scale

There are many scales to measure work–family conflict. The scale selected in this part is a work–family conflict measurement tool specially developed by Haslam et al. (2014) for children’s parents. The Work–Family Conflict Scale (WFCS) consists of 10 items and assesses two forms of work–family conflict, the work-to-family conflict (WFC; 5 items) and the family-to-work conflict (FWC; 5 items). Work-to-family conflict inquired how work demands interfere with family responsibilities (e.g., “To what extent do you think ‘My work prevents me spending sufficient quality time with my family’? “). Family-to-work conflict asked whether family engagement interferes with work performance (e.g., “To what extent do you think ‘My work performance suffers because of my personal and family commitments’? “). This study focused on examining the impact of work-to-family conflict on family relationships and child development, so only the dimension of work-to-family conflict was measured. Items are assessed on a 7-point Likert scale ranging from one (completely disagree) to seven (completely agree). There was no reverse scoring item in the scale, and the higher the score, the more serious the work-to-family conflict parents face. Jung and Kim (2021) have examined the psychometric properties of the Chinese version of the WFCS, results showed that the scale had concurrent and discriminant validity. At the same time, the internal consistency reliability for the work-to-family conflict subscale was examined in Jung and Kim’ research, the Cronbach’s α coefficient of the WFC subscale was 0.87.

### Social competence and behavior evaluation scale

This study chose the Social Competence and Behavior Evaluation Scale (SCBE-30) compiled by LaFreniere and Dumas (1996) to measure the preschool children’s social behavior problems. This scale contains a total of 30 items, using a 6-point Likert scale ranging from one (never) to seven (always), reported by the preschool children’s parents. There are three dimensions of social competence (SC), anger-aggression (AA), and anxiety-withdrawal (AW) in the scale. This study intends to only measure preschool children’s social behavior problems, therefore, two dimensions AA and AW are selected, and the higher the score on each dimension, the more common the preschool children’s social behavior problems. Liu et al. (2011) conducted an internal consistency reliability analysis on the above two factors, and the Cronbach’s α coefficients of AA and AW were 0.66 and 0.81 respectively, which met the requirements of psychometrics. The results of confirmatory factor analysis showed that SCBE-30 has a good structure and construct validity (*χ^2^/df* = 2.48, GFI = 0.880, NFI = 0.870, NNFI = 0.910, CFI = 0.920, RMSEA = 0.060; Liu et al., 2011). It proves that the SCBE-30 can be used as an effective tool to measure the social behavior problems of Chinese children.

**Table 1:** Demographic characteristics of the sample.

	Fothers (*N* = 590; 19.4%)	Mothers (*N* = 2,448; 80.6%)	Totle (*N* = 3,038; 100%)
Level of education			
High school or lower	93 (15.8%)	483 (19.7%)	576 (19.0%)
Junior college education	129 (21.9%)	699 (28.6%)	828 (27.3%)
Bachelor’s degree	305 (51.7%)	1,086 (44.4%)	1,391 (45.8%)
Master’s degree or higher	63 (10.7%)	180 (7.4%)	243 (8.0%)
Have the only child or not			
Yes	157 (26.6%)	659 (26.9%)	816 (26.9%)
No	433 (73.4%)	1,789 (73.1%)	2,222 (73.1%)
Child gender			
Boy	304 (51.5%)	1,243 (50.8%)	1,547 (50.9%)
Girl	286 (48.5%)	1,205 (49.2%)	1,491 (49.1%)
Child age			
3 years old	156 (26.4%)	608 (24.8%)	764 (25.1%)
4 years old	166 (28.1%)	756 (30.9%)	922 (30.3%)
5 years old	205 (34.7%)	844 (34.5%)	1,049 (34.5%)
6 years old	63 (10.7%)	240 (9.8%)	303 (10.0%)
Single-parent family or not			
Yes	19 (3.2%)	60 (2.5%)	79 (2.6%)
No	571 (96.8%)	2,388 (97.5%)	2,959 (97.4%)

Percentages may not add up to 100 due to missing data.

### Guilt about parenting scale

This study used the Guilt about Parenting Scale (GAPS), a unidimensional structured scale consisting of 10 items developed by Haslam and Finch (2016). The scale is scored on a 7-point Likert scale from one (very strongly disagree) to seven (very strongly agree), with higher scores indicating higher levels of guilt about parenting experienced by parents. Examples of typical items in this scale include statements such as “I often worry I am not as good a parent as I should be,” “I often feel it is my fault if my child gets upset,” and similar phrases. The Cronbach’s α of GAPS in Chinese was 0.89, the internal consistency reliability and retest reliability of the scale met the measurement requirements, indicating that the scale has high reliability and stability across time. Zeng et al. (2021) confirmed that the one-way model fits well (*χ^2^/df* = 3. 17, CFI = 0. 95, TLI = 0. 93, RMSEA = 0. 07, SRMR = 0. 04).

### Parenting and family adjustment scale

The parent–child relationships was a factor in the Parenting and Family Adjustment Scale (PAFAS; Sanders et al., 2014). The whole scale is a 30-item measure for parents of children aged 2 to 12 years. The parent–child relationships part of the PAFAS consists of 5 items, items are rated on a 4-point Likert scale ranging from one (not true of me at all) to four (true of me very much), they are summed to provide overall scores, with higher scores indicating higher levels of parent–child relationships. Sanders et al. (2014) have demonstrated good psychometric properties of PAFAS in an Australian sample, the construct and concurrent validity as well as internal consistency of this measure are satisfactory (coefficients ranged from 0.70 to 0.87). Guo et al. (2017) validated the PAFAS in a Chinese cultural context, the results of their study “demonstrated that PAFAS had satisfactory construct validity, acceptable internal reliability, and excellent convergent and concurrent validities in a Chinese sample.” Therefore, PAFAS can be an effective and satisfactory tool in the present study. Only parent–child relationships part was used in this study, its *H* coefficient was 0.82 (Guo et al., 2017).

### Validity and reliability test

The present study employed SMART-PLS software to evaluate and examine the data of the measurement model and structural equation model. Specifically, this included evaluating the reliability of consistency, composite reliability, convergent validity, and discriminant validity. Cronbach’s alpha coefficient and composite reliability (CR) were selected to analyze the reliability of the questionnaire in this study. As shown in Table 2, the Cronbach’s α value for each variable ranged from 0.852 to 0.931, while the CR values ranged from 0.864 to 0.948. All the values exceeded 0.8, indicating a high level of reliability for the scales.

**Table 2:** Validity and reliability test.

	Cronbach’s Alpha	rho_A	Composite reliability	Average variance extracted
GP_	0.852	0.833	0.864	0.423
PCR_	0.873	0.878	0.908	0.663
PSBP_	0.865	0.868	0.887	0.506
WFC	0.931	0.937	0.948	0.785

Validity test is the process of confirming whether a measurement tool accurately measures the intended construct. Validity analysis of the model typically examines both content validity and structural validity. In terms of content validity, the variable items designed in this study were derived from relevant literature and underwent multiple reviews by domain experts. Additionally, a pilot study was conducted, and the scales were subsequently revised based on the findings. Thus, it can be concluded that the scales possess high level of content validity. Regarding structural validity, convergent validity is commonly assessed using the Average Variance Extracted (AVE) criterion. In this study, the AVE values of three variables exceeded 0.5, and the AVE value of only one variable was lower than 0.5 but close to 0.5, indicating good convergent validity for the scales.

This study assessed discriminant validity using the Fornell-Larcker criterion. When AVE value of each variable is greater than its squared correlation with other variables, it indicates good discriminant validity in the latent variable model. As depicted in Table 3, the square root of AVE for each latent variable ranged from 0.553 to 0.886, all exceeding the correlations with other latent variables. This indicates that the model possesses good discriminant validity.

**Table 3:** Discriminant validity test.

	GP_	PCR_	PSBP_	WFC
GP_	0.651			
PCR_	−0.071	0.814		
PSBP_	0.236	−0.345	0.553	
WFC	0.382	−0.143	0.246	0.886

Furthermore, this study employed the Variance Inflation Factor (VIF) method to examine the presence of multicollinearity in the sample data. The VIF test results indicated a maximum value of 4.707, a minimum value of 1.228, and a mean value of 1.955, all of which were below the threshold of 10 for multicollinearity. Therefore, there is no issue of multicollinearity among the variables in this study.

## Research results

This study first calculated descriptive statistics for all variables using SPSS version 23.0 to estimate the mean, standardized deviation between the main variables, and tested the two-way correlations between work-to-family conflict, preschool children’s social behavior problems, guilt about parenting and parent–child relationships by calculating Pearson’s coefficient. And then, controlling for demographic variables such as “female or male” “Only child or not” and “whether grandparents are involved in parenting” the mediating effects of guilt about parenting and parent–child relationships on work-to-family conflict and preschool children’s social behavior problems were analyzed using SPSS PROCESS Model 6. PROCESS is a plugin specifically designed for analyzing mediation and moderation effects. It can be used with traditional data analysis software such as SPSS and SAS. PROCESS simplifies the multiple steps required for traditional mediation analysis into a single step, automatically conducting Bootstrap tests for mediation effects. It is scientifically robust, efficient, and user-friendly. Therefore, this study employed SPSS PROCESS Model 6 for model construction and path analysis. The statistical results, including path coefficients, confidence intervals, significance etc., were used to explain the direction and significance of the mediating effects. Besides, this study used Full Information Maximizing-Likelihood (FIML) to process the missing data in the statistical analyzes.

### Common method variance analysis

Given that the data was obtained through subjective self-reports from the parents of the children, there is a possibility of common method bias. Based on the suggestions of Zhou and Long (2004), the data collection process of this study was controlled in terms of measurement procedures, such as the use of anonymous forms for the measurement and the use of some reverse questions. After completing the data collection, Harman’s single factor test was conducted on the study variables (Harman, 1976). Three factors had eigenvalues greater than one, as shown by the findings. The first factor explained 35.7% of total variation, less than the 40% threshold criterion (Podsakoff et al., 2003), indicating that no significant common method bias existed.

### Descriptive and correlation analyzes

Table 4 provides descriptive and correlation analyzes of the four variables: work-to-family conflict, guilt about parenting, parent–child relationships, and preschool children’s social behavior problems. Pearson’s product–moment correlation coefficient was employed in this study to investigate the associations between the primary variables of interest. The results indicated significant correlations between work-to-family conflict, guilt about parenting, and social behavior problems in preschool children. Additionally, there was a significant correlation among work-to-family conflict, guilt about parenting, parent–child relationships, and social behavior problems in preschool children, with correlation coefficients ranging from −0.298 to 0.332. There was a statistically significant correlation between the variables.

**Table 4:** Means standard deviations and correlations of variable (*n* = 3,808).

Variables	M	SD	1	2	3	4
1. Work-to-family conflict	17.51	7.10	1			
2. Guilt about Parenting (GP)	45.54	10.09	0.332^***^	1		
3. Parent–child Relationships (PCR)	17.55	2.68	−0.140^***^	0.041^**^	1	
4. Preschoolers’ Social Behavior problems (PSBP)	38.57	7.45	0.231^***^	0.126^***^	−0.298^***^	1

^***^p < 0.001, ^**^p < 0.01. All tests were two-tailed.

### Chain mediation model analysis

This study used a mediation model to explore the effects of work-to-family conflict and preschool children’s social behavior problems. To examine the mediating impact, the non-parametric percentile bootstrapping method with bias correction was utilized. For the analysis, the bootstrapping method with 5,000 subsamples was adopted. Table 5 revealed the SEM path coefficients. The *R*^2^ coefficient is a measure of the goodness-of-fit of the structural model and indicates the explanatory power of the endogenous latent variables. It ranges from 0 to 1, with higher values indicating better predictive capabilities. As demonstrated in Table 5, the explanatory power of the structural model in this study was found to be 11, 2.8, 13.2, and 5.3% for the respective variables. These values achieved statistical significance, indicating a meaningful level of explanatory power.

**Table 5:** SEM path coefficients.

	Standardized			*R* ^2^	*F*
	*β*	*SE*	*t*		
Work-to-family conflict to guilt about parenting	0.473^***^	0.024	19.406	0.110^***^	376.587
Work-to-family conflict to parent–child relationships	−0.065^***^	−0.007	−9.071	0.028^***^	43.740
Guilt about parenting to parent–child relationships	0.026^***^	0.005	5.161		
Work-to-family conflict to preschoolers’ social behavior problems	0.173^***^	0.019	45.194	0.132^***^	153.176
Guilt about parenting to preschoolers’ social behavior problems	0.061^***^	0.013	4.569		
Parent–child relationships to preschoolers’ social behavior problems	−0.775^***^	0.048	−16.244		
Work-to-family conflict to guilt about parenting to parent–child relationships to preschoolers’ social behavior problems	0.242^***^	0.019	13.076	0.053^***^	170.968

^***^p < 0.001, ^**^p < 0.01.

The results showed that work-to-family conflict had a significant predictive effect on guilt about parenting (*β* = 0.473, *p* < 0.001), parent–child relationships (*β* = −0.065, *p* < 0.001) and preschool children’s social behavior problems (*β* = 0.173, *p* < 0.001). Guilt about parenting significantly predicted parent–child relationships (*β* = 0.026, *p* < 0.001) and preschool children’s social behavior problems (*β* = 0.061, *p* < 0.001). Parent–child relationships significantly predicted preschool children’s social behavior problems (*β* = −0.775, *p* < 0.001). In addition, the complete path from “work-to-family conflict” to “guilt about parenting” to “parent–child relationship” to “preschoolers’ social behavior problems” also has a significant predictive effect (*β* = 0.242, *p* < 0.001).

**Figure 2:**
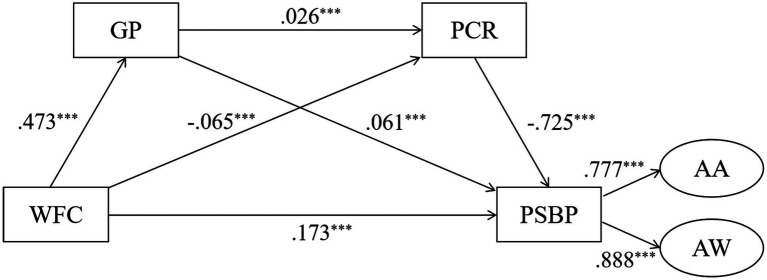
The chain mediation model, ^***^p < 0.001, ^**^p < 0.01. WFC, Work-to-Family Conflict; PSBP, Preschoolers’ Social Behavior Problems; AA, Anger-aggression; AW, Anxiety-withdrawal; GP, Guilt about Parenting; PCR, Parent–child Relationships.

Table 6 showed the mediating effects of guilt about parenting and parent–child relationships between parents’ work-to-family conflict and preschool children’s social behavior problems. Figure 2 was a chain mediating model. The direct effect of parents’ experienced work-to-family conflict on preschool children’s social behavior problems was 0.136 [95% *CI:* (0.114, 0.155)]. To further verify the mediating effect of guilt about parenting and parent–child relationships on work-to-family conflict and preschool children’s social behavior problems, the results ranged from 0.026 to 0.775 (95% *CI*, not including zero), demonstrating that guilt about parenting and parent–child relationships mediated the relation between parents’ work-to-family conflict and preschool children’s social behavior problems, and the total standardized mediating effect value was 0.242 [95% *CI:* (0.206, 0.279)]. Particularly, the mediating effect comprised three indirect effects, namely, path 1: work-to-family conflict → guilt about parenting → preschool children’s social behavior problems (0.029); path 2: work-to-family conflict → parent–child relationships → preschool children’s social behavior problems (0.050); and path 3: work-to-family conflict → guilt about parenting → parent–child relationships → preschool children’s social behavior problems (−0.009). The ratios of the three indirect effects to the total effect were 12.0, 20.7, and 3.7 for path 1, 2, and 3, respectively. Hypotheses 2, 3, and 4 could be confirmed. Furthermore, we compared the indirect effects of different paths in pairs to examine if there were significant path differences. In comparison 1, the bootstrap 95% confidence interval for the difference between indirect effects 1 and 2 did not include 0 [95% *CI:* (−0.040, −0.003)], representing a statistically significant difference between the two. By the same method of comparison, there were significant difference between indirect effects 1 and 3 [comparison 2, 95% *CI:* (0.024, 0.053)] or between indirect effects 2 and 3 [comparison 3, 95% *CI:* (0.046, 0.074)].

**Table 6:** Guilt about parenting and parent–child relationships in the mediating effect analysis.

Effect	Path relationship	Effect size	Bootstrap 95% CI	Relative mediation effect
Direct effect	Work-to-family conflict - > Preschoolers’ social behavior problems	0.173	[0.135, 0.210]	0.710
Path1	Work-to-family conflict - > Guilt about parenting - > Preschoolers’ social behavior problems	0.029	[0.015, 0.042]	0.120
Path2	Work-to-family conflict - > Parent–child relationships - > preschoolers’ social behavior problems	0.050	[0.038, 0.063]	0.207
Path3	Work-to-family conflict - > Guilt about parenting - > Parent–child relationships - > Preschoolers’ social behavior problems	−0.009	[−0.013, –0.006]	−0.037
Total effect		0.242	[0.206, 0.279]	1

In order to validate the effectiveness of the model and mitigate endogeneity issues, this study employed the method of variable substitution to examine the robustness of the model. Measures of parent–child relationships were used to assess the interactive characteristics between parents and preschool children. Given the close association between parenting styles and parent–child relationships (e.g., Duncan et al., 2009; Siu et al., 2016), parenting styles serve as a direct reflection of the interactive dynamics between parents and children. Hence, we substituted the mediating variable “parent–child relationships” with “parenting styles” and employed the same modeling approach for measurement. The findings revealed a favorable model fit (*R*^2^ = 0.053, *F* = 170.969, *β* = 0.243, *t* = 13.076, *p* < 0.001) with statistical significance, indicating a high level of model robustness and replicability.

## Discussion

This study contributes to the existing literature by investigating the underlying mechanisms linking work-to-family conflict and social behavior problems in preschool-aged children. The study was conducted with a sample of parents of preschool children residing in several developed coastal cities in Guangdong Province, China. We utilized a chain mediation model to test the hypotheses, whereby guilt about parenting mediated the association between work-to-family conflict and preschool children’s social behavior problems, and parent–child relationships mediated the same association. Furthermore, we observed that work-to-family conflict might indirectly influence preschool children’s social behavior problems through the chain mediating effect of guilt about parenting and parent–child relationships. Collectively, these findings provide a comprehensive and constructive perspective on the relationship between work-to-family conflict and social behavior problems in preschool-aged children.

### Association between work-to-family conflict and preschool children’s social behavior problems

This study found that work-to-family conflict of parents was positively associated with preschool children’s social behavior problems. Work-to-family conflict can positively predict children’s anger-aggression and anxiety-withdrawal. This result validated the hypothesis 1 of this study, indicating that work-to-family conflict of parents is strongly associated with different aspects of preschool children’s social behavior problems, and is generally consistent with the results of previous studies (Matias et al., 2017; Yucel and Latshaw, 2018; Chai and Schieman, 2021). The ecological theory of child development suggests that work-family relationship is an important part of the child development system. In the Chinese cultural context, the ages from 31 to 40 is the rising period of career development for most employees (Li et al., 2012). On the one hand, they tend to put in more experience and effort to pursue rapid career growth. On the other hand, in the eyes of work leaders, this age group is seen as “young and strong” and has some work experience (capable of doing their current job), so leaders tend to give them more work assignments. For parents of preschool children, they expect or are asked to put in a lot of work effort, while at the same time, young children aged 3–6 years old need a lot of parenting and support. Parents often play different roles and shift roles in work and family spaces, and when the roles in a given domain are required to expand, the boundaries between the two are displaced and inter-role conflicts arise (Clark, 2000). When work interferes with family, it is an important source of stress for parents that can influence an individuals’ well-being (Frone et al., 1994). For both male and female, high level of work-to-family conflict reduces parents’ life satisfaction, triggers more psychological stress, higher risk of burnout and higher depression (Kossek and Ozeki, 1998; McNall et al., 2010; Aarntzen, 2020). Parents’ high level of pressure will be transferred to parenting behavior and emotional expression, which will virtually create a high-stress family atmosphere for young children. In this atmosphere, young children will internalize the pressure they perceive, which will lead to social problems such as anxiety and withdrawal. In conclusion, the daily stressful state of parents, as one of the important psychological micro-environments for young children, may directly cause anger-aggression, anxiety-withdrawal, and other undesirable tendencies in preschool children’s social interactions.

Finally, it is important to note that some studies have found that the more time employees spend on themselves, the less conflict they experience between work and family (Spell et al., 2009). A few findings have been consistent in suggesting that hyperactive, aggressive, or noncompliant children require more parents’ attention, and parents have to devote more time to the family and less time to themselves, which in turn reinforces work-to-family conflict. It is clear that difficult children often elicit more inconsistent and aggravated behaviors from mothers (Bates and Bayles, 1988; Campbell et al., 1991; Shaw et al., 1994), preschool children’s social behavior problems play a reaction role to work-to-family conflict.

### Mediating role of guilt about parenting

This study found that work-to-family conflict of male and female can affect preschool children’s social behavior problems through guilt about parenting, which is basically consistent with our hypothesis 2. That is guilt about parenting mediated the association between work-to-family conflict and preschool children’s social behavior problems. In terms of the first stage of the mediation link from work-to-family conflict to guilt about parenting, work-to-family conflict is positively associated with guilt about parenting, which supports the findings of previous studies. Balancing different life roles can be even more difficult for parents of preschool children, who are prone to feelings of guilt about parenting (Martínez et al., 2011; Borelli et al., 2016; Korabik, 2017). Work demands and characteristics have direct impacts on employees’ family lives, and guilt about parenting arise when parents feel they have violated the standards of family education (Klass, 1987; Jones and Kugler, 1993).

The second stage of the mediation link is the predictive effect of guilt about parenting on preschool children’s social behavior problems. From the point of view of the direct connection from guilt about parenting to preschool children’s social behavior problems, this study found guilt about parenting is detrimental, hindering the healthy development of children’s social adjustment. For example, the guilt from work-to-family conflict may be detected by children, inadvertently conveying negative information about the meaning of parental employment, thereby influencing children’s behavior and triggering their social behavior problems. Therefore, parents with higher work-to-family conflict tend to have higher guilt about parenting, and then their children’s social adjustment can be disturbed, behavior problems will increase.

### Mediating role of parent–child relationships

The results of this study also showed that parent–child relationships played a mediating role between work-to-family conflict and preschool children’s social behavior problems. The work-to-family conflict could do harm to preschool children’s social behavior by breaching parent–child relationships, which was consistent with our hypothesis 3 and supported ecological systems theory and attachment theory (Bowlby, 1979). “When parents experience difficulties with excessive work demands—a characteristic in children’s exosystem—they might not be able to fully monitor and fulfill the needs of children, which can negatively affect parental role functioning” (Chai and Schieman, 2021). It is not difficult to deduce that when work interferes with the time, emotions and energy parents devote to family education, it directly affects parents’ attitudes toward their preschool children, social behavior parenting and guidance, and parent–child relationships, leading to more social behavior problems (Cho and Allen, 2012; Jung and Kim, 2021). Early childhood is a sensitive period for interpersonal relationships. Popov and Ilesanmi (2015) emphasized that only the children that manage to have a good relationship with their parents will extend social and emotional relationships normally with their peers. Many studies in developmental psychopathology have shown that parent–child relationships in early childhood are an important factor affecting the development of behavior problems in children (Masten and Garmezy, 1985; Galen and Underwood, 1997; Schmeck and Poustka, 2001). This study reconfirmed the conclusions of most previous studies in this field, that is, work-to-family conflict will affect the harmony and intimacy of parent–child relationships, and interfere with the creation of a good parent–child relationships and family atmosphere, thereby affecting the psychological development of children and leading to social behavior problems.

### Chain mediating role of guilt about parenting and parent-child relationships

This study showed that work-to-family conflict act on the preschool children’s social behavior problems through the chain mediation effect of guilt about parenting and parent–child relationships, and guilt about parenting significantly positively predicts parent–child relationships, which is similar to the conclusions of previous studies (Tangney, 1990; Leith and Baumeister, 1998). This result also proved the related viewpoints of the three theories: the ecosystem of child development theory, spillover-crossover model of emotion, and attachment theory. Guilt about parenting is the emotional set of parents based on the evaluation of parenting behavior and results, and it is an important individual factor of parents in the theoretical hypothesis of this study. Specifically, when parents experience work-to-family conflict, they are more likely to feel guilty about their children and to adjust their time and energy so as to ensure better parent–child relationships. Past studies have shown that guilt tendencies can promote individuals to think about their roles, evaluate the responsibilities of different social roles, and gain motivation to take corrective measures when faced with role conflicts (Tangney, 1990; Leith and Baumeister, 1998). The constructive nature of guilt suggests that guilt may alleviate the negative relationship between work-to-family conflict and parent–child interactive behaviors. In other words, guilt about parenting promotes parents to reflect and adjust the parenting time, investment and specific practices, and then reduces the possibility of work-to-family conflict causing children’s social adjustment problems by improving the parent–child relationships.

It can be seen that the influence of guilt about parenting on children’s psychological growth has two sides. First, guilt about parenting is a negative emotion that may be detected by children, inadvertently conveying negative messages about the meaning of parents’ employment, which could influence children’s behavior (Borelli et al., 2016). However, some studies have also found that guilt about parenting might have not decreased the level of parent–child relationships, as the parents assumed responsibilities for work-to-family conflict and had higher motivation to take corrective actions. Parents’ self-evaluation and guilt based on correct parenting concepts, as well as the resulting adjustment of parenting behaviors, may reduce the negative impact of external adverse objective environments on children’s growth, (e.g., insufficient economic income, parents’ low educational level, work-to-family conflicts, etc). This is a typical manifestation of parents’ emotions and behaviors mitigating the negative impact of the external adverse environment on children’s growth.

In summary, the emotional experience of guilt about parenting, when not accompanied by corresponding behavioral changes, can exacerbate parental stress and anxiety, leading to problem behaviors in children. On the other hand, when parents actively adjust their parenting behaviors in response to feelings of guilt (such as adopting permissive parenting styles or increasing parental involvement), it may lead to an improvement in the parent–child relationships. The current study proposes a chain mediation model wherein guilt about parenting plays a corrective role by reducing the impact of work-to-family conflict on active parent–child activities.

## Conclusion

Social skill development is critical to children’s psychological and physical development. This study investigated the underlying mechanisms accounting for the associations between work-to-family conflict and preschool children’s social behavior problems. Work-to-family conflict is a form of inter-role conflict that occurs due to a shortage of resources (e.g., time, energy, or emotion) that makes it difficult to successfully perform in the family domain. This study suggested that work-to-family conflict could make a difference to preschool children’s social behavior problems through a separate indirect path via guilt about parenting or parent–child relationships. Meanwhile, work-to-family conflict may also be associated with preschool children’s social behavior problems through the chain mediating effect of guilt about parenting and parent–child relationships. These findings provide a theoretical foundation for reducing the negative impact of parents’ work-to-family dilemmas on children’s healthy growth, as well as some practical guidance for social security and kindergarten education in promoting the healthy growth of children under the tripartite cooperation between family, kindergarten and society.

## Limitations and implications

This study has several limitations that need to be addressed. Firstly, the research design is cross-sectional design, which precludes any causal inferences regarding the relationship between different variables. To overcome this limitation, future studies could employ a longitudinal design to track the developmental trajectory of children’s adaptability and behavior problems and verify the long-term impact of parents’ work-to-family conflict, guilt about parenting, and parent–child relationships on children’s development. Secondly, the data in this study were solely based on self-reports from parents of preschool children. To mitigate measurement bias, future studies could employ multi-subject reports, such as evaluations of parenting processes and preschool children’s social characteristics from kindergarten teachers. Moreover, intervention studies on parents’ work-to-family conflict could be conducted to verify whether such conflict is conducive to the development of children’s social adaptation behavior.

Although there are some limitations, this study still holds both theoretical and practical contribution. While previous studies have mainly focused on the impact of work-to-family conflict on parents’ personal health and their children’s well-being, few studies have specifically investigated the relationship between work-to-family conflict and children’s social behavior problems, particularly within the context of Chinese economic and cultural factors that shape parents’ work demands and parenting philosophies. By exploring the emotions that Chinese parents experience when faced with work-to-family conflict and how they mitigate its effects on their children, this study fills this gap in the literature. These findings in this study highlight the important role of work-to-family conflict, parenting guilt, and parent–child relationships on preschool children’s social behavior problems, and further illuminate the chain mediating role of guilt about parenting and parent–child relationships. These results are consistent with the ecological system theory and attachment theory and have practical implications for enhancing parents’ educational engagement and promoting children’s growth.

Based on the findings, this study concluded that family education for preschool children is not solely the responsibility of parents, but rather requires coordination among all social actors to balance work and family demands. To this end, support and protection of children’s family education at the social level should be strengthened, efforts should be made to ensure adequate childcare resources, active guidance in childcare concepts and methods, and stage-by-stage follow-up assessments of children’s social development should be conducted.

## Data availability statement

The original contributions presented in the study are included in the article/supplementary material, further inquiries can be directed to the corresponding authors.

## Ethics statement

The studies involving human participants were reviewed and approved by Minzu university of China. The patients/participants provided their written informed consent to participate in this study.

## Author contributions

YW conceptualized the study, supervised the data collection and processing, and provided critical feedback on all versions of the manuscript. DS was responsible for securing research funding and provided input in all stages of the research process. YW, GL, and XZ worked together to draft the introduction, results, and discussion sections, and provided extensive data to support the presented ideas. MZ reviewed the data analysis, composed the data analysis section, and thoroughly revised the entire manuscript. All authors contributed to the article and approved the submitted version.

## Conflict of interest

The authors declare that the research was conducted in the absence of any commercial or financial relationships that could be construed as a potential conflict of interest.

## Publisher’s note

All claims expressed in this article are solely those of the authors and do not necessarily represent those of their affiliated organizations, or those of the publisher, the editors and the reviewers. Any product that may be evaluated in this article, or claim that may be made by its manufacturer, is not guaranteed or endorsed by the publisher.

## Footnotes

^1^"Preschool stage" include ages from 3 to 6 years in China, it’s a time range of children’s kindergarten life from the beginning to the end.
